# Differentiating mathematical mindset, growth mindset, and self-efficacy through intervention research: a neuroplasticity approach

**DOI:** 10.3389/fpsyg.2025.1598817

**Published:** 2025-06-05

**Authors:** Xiaoyu Xu, Jack A. Dieckmann

**Affiliations:** ^1^School of Mathematics, Guangdong University of Education, Guangzhou, Guangdong, China; ^2^Graduate School of Education, Stanford University, Stanford, CA, United States

**Keywords:** mindset, mathematical mindset, growth mindset, self-efficacy, mathematics performance

## Abstract

Mathematical Mindset (MM), Growth Mindset (GM), and Self-Efficacy (SE) are critical psychological constructs that shape students' mathematical achievement by influencing cognitive flexibility, problem-solving strategies, and motivational persistence. This study, based on data derived from Xu et al., extends prior research by examining the distinct contributions of MM, GM, and SE in response to an intervention among university students. To better understand how neuroplasticity, MM, and GM manifest in this study, thematic analysis was applied to qualitative interview data, providing deeper insights into the cognitive and behavioral changes induced by the intervention. Using a mixed-methods approach, we analyze quantitative data through correlation heatmaps, contour plots, and scatter visualizations, alongside qualitative data, to provide a comprehensive understanding of how these constructs interact. Findings reveal that MM and SE exhibit a synergistic relationship, where higher levels of both correspond to increased academic performance, cognitive adaptability, and engagement in mathematics. GM, while indirectly influencing achievement, primarily enhances perseverance and learning from mistakes, contributing to the reinforcement of MM and SE. Future research should refine the measurement of MM and GM across different learning environments, investigate the transferability of GM across domains, and explore the role of brain activity in optimizing intervention effectiveness.

## 1 Introduction

Cognitive development is vital in students’ achievements, in mathematics, where the brain’s ability to adapt and form new neural connections—known as neuroplasticity (NP)—is essential for acquiring and applying mathematical learning (Ansari, 2015; Moser et al., 2011). Several studies emphasize the role of neuroplasticity in fostering cognitive flexibility, with the Growth Mindset (GM)—the belief that intelligence and abilities can be developed through effort and persistence—is widely recognized for its ability to enhance students’ motivation, resilience, and their capacity to embrace challenges, learn from mistakes, and persist in the face of difficulties, ultimately leading to improved academic outcomes (Blackwell et al., 2007; Dweck, 2006a, b). In contrast, a Fixed Mindset views intelligence as static and unchangeable, leading students to become discouraged by challenges or setbacks (Dweck, 2006a; Blackwell et al., 2007). Growth mindset is embedded within a broader framework known as Meaning Systems, which includes key psychological factors such as goal orientations (performance vs. learning goals), beliefs about effort, and attribution styles (Yeager and Dweck, 2020). These interconnected elements shape individuals’ attitudes toward learning, influencing their motivation, persistence, and responses to challenges. Applied to mathematics learning, this concept has demonstrated significant positive outcomes, enhancing students’ motivation, resilience, and engagement with challenging tasks, while interventions aimed at cultivating a growth mindset have been linked to increased neural plasticity, particularly in brain regions associated with learning and memory, such as the prefrontal cortex and hippocampus (Dweck, 2006a,b; Blackwell et al., 2007; Moser et al., 2011; Ansari, 2015).

Rooted in the principles of GM (Dweck, 2006a,b), a Mathematical Mindset (MM) encourages students to view mathematics as a creative, exploratory subject rather than a fixed set of rules or procedures. MM emphasizes the belief that everyone can excel in math through effort, practice, and a positive attitude. According to Jo Boaler, MM involves embracing challenges, learning from mistakes, and understanding that mathematical ability is not innate but can be developed through persistence and effective strategies (Boaler, 2016). This mindset fosters deeper engagement with mathematical concepts, enhances problem-solving skills, and helps students build resilience and confidence in their mathematical abilities. While Mathematical Mindset (MM) and Growth Mindset (GM) have shown promise in enhancing motivation and resilience in mathematics, their ability to fully account for academic success remains incomplete. For instance, although GM promotes the belief that abilities can improve through effort, research indicates this mindset does not always translate to performance gains (Claro et al., 2016; Rattan et al., 2012), with factors such as students’ perceived effort, contextual understanding, and access to support systems critically shaping outcomes. Similarly, while MM’s framing of mathematics as a creative, exploratory discipline (Boaler, 2016) holds value, it may struggle to overcome entrenched academic barriers or sustain long-term engagement among students with diverse learning backgrounds and challenges (Kelly et al., 2020). Existing research has primarily examined the isolated effects of Mathematical Mindsets (MM), Growth Mindsets (GM), and Self-Efficacy (SE) in K-12 settings (Boaler, 2016; Claro et al., 2016). However, a significant gap remains in understanding how these constructs interact dynamically to shape learning outcomes in university students—a population facing distinct cognitive, social, and institutional challenges. For instance, unlike K-12 learners, university students’ SE is closely tied to disciplinary identity formation (e.g., “being a mathematician”; Sahagun et al., 2021), which may influence the interplay between MM and GM. Additionally, institutional factors such as gendered stereotypes in STEM disproportionately impact SE among marginalized university students (Cort et al., 2020), yet research has not explored whether MM/GM interventions can mitigate these effects. Indeed, previous literature has consistently documented gender as an influential factor shaping students’ mathematical attitudes, self-efficacy, and engagement, suggesting that gender could meaningfully contextualize mindset-based interventions (Gunderson et al., 2013; Wang and Degol, 2017). This raises an important point: mindset may be just as crucial for university students as it is for younger learners. Emerging evidence from freshman-level interventions, including Xu et al. (2022) demonstration of the efficacy of Mathematical Mindset interventions during critical transitional periods, suggests that mindset training can help bridge transitional challenges. Building on this, there is a critical need for a more comprehensive analysis of how MM and GM influence the academic trajectories of university students.

Emerging empirical work demonstrates that growth mindset (GM) interventions not only reshape learners’ attribution of setbacks (Zhao H. et al., 2023; Yeager and Dweck, 2023) but also amplify domain-specific self-efficacy (SE) through neuroplastic reinforcement of effort-to-mastery pathways, a mechanism validated in both behavioral experiments and error-monitoring system studies (Chen et al., 2022). This GM-SE synergy has been shown to stabilize academic persistence under high cognitive demand, particularly in mathematics (Hwang and Son, 2021).Students with high self-efficacy are more likely to set ambitious goals, exert greater effort, and demonstrate resilience in the face of difficulties (Pajares and Miller, 1994). In the mathematical domain, students who believe in their problem-solving abilities are more likely to approach complex tasks with confidence and adaptability. Moreover, self-efficacy (SE) is dynamic—it can be developed and strengthened through mastery experiences, positive reinforcement, and the observation of successful peers (Bandura, 1997), which further enhances their capacity to tackle challenging problems. Therefore, by fostering both a Mathematical Mindset (MM) and high Self-Efficacy (SE), educators can create a robust learning environment that not only encourages academic achievement but also nurtures students’ long-term motivation and active engagement with mathematics. These psychological constructs are interconnected and mutually reinforcing such as goal setting. For instance, a growth mindset can enhance self-efficacy by encouraging students to believe in their ability to improve through effort, and in turn, self-efficacy can strengthen a growth mindset by providing evidence of progress (Schunk and Pajares, 2009).

Building on Xu et al. (2025), we adopt the full set of intervention designs and utilize Xu’s data for analysis. Together, these constructs—Growth Mindset (GM), Mathematical Mindset (MM), and Self-Efficacy (SE)— collectively contribute to the development of a positive mathematical mindset that enhances learning and strengthens resilience in mathematics. The research questions (RQ) guiding this investigation are as follows:

*RQ 1*: What is the relative contribution of Mathematical Mindset and Self-Efficacy to changes in students’ mathematics performance following an intervention?

*RQ 2*: What are the relationships among Growth Mindset, Mathematical Mindset, and Self-Efficacy, and how do they relate to students’ neurocognitive and behavioral responses following the intervention?

## 2 Literature review

### 2.1 Neuroplasticity and learning: the brain’s adaptability

Neuroplasticity fundamentally shapes cognitive and behavioral outcomes in mathematics through dynamic interactions between neural adaptation and learning experiences. Enhanced connectivity between the anterior cingulate cortex (ACC) and dorsolateral prefrontal cortex (DLPFC) underpins error-driven adaptation, a process critical for refining problem-solving strategies. Students who perceive mistakes as opportunities for growth exhibit amplified ACC activation during error monitoring, which correlates with faster corrective adjustments and reduced error repetition rates (Schroder et al., 2017; Moser et al., 2011). This aligns with findings from Holroyd and Coles (2002), who identified the error-related negativity (ERN) as a neural marker of adaptive learning, suggesting that growth mindset interventions may amplify ERN magnitude to optimize error processing (Dweck, 2008). Longitudinal studies further demonstrate that error-reframing training increases ACC gray matter density over 8 weeks, directly linking neuroplastic changes to improved algebraic reasoning (Sarrasin et al., 2018; Yeung et al., 2006).

Working memory optimization emerges as another hallmark of neuroplasticity, driven by parietal lobe plasticity—particularly in the intraparietal sulcus (IPS). Adaptive mathematical training strengthens visuospatial and symbolic processing networks, with children showing increased IPS activation during mental arithmetic tasks (Kucian and Kaufmann, 2009; De Smedt et al., 2010). Such plasticity is behaviorally measurable: students exposed to IPS-focused interventions outperform peers in working memory span tests, mediated by enhanced functional connectivity between the IPS and prefrontal regions (Supekar et al., 2013; Klingberg, 2010). For instance, Klingberg (2010) working memory training paradigm improved both IPS activation and arithmetic fluency, underscoring the bidirectional relationship between neural adaptation and skill acquisition.

Equally critical is neuroplasticity’s role in anxiety modulation. Math-specific stress-inoculation training reduces amygdala hyperactivity, paired with declines in self-reported anxiety (Young et al., 2012; Ashcraft and Krause, 2007). These neural shifts predict long-term behavioral outcomes, including increased enrollment in advanced math courses (Lyons and Beilock, 2012). Pharmacological studies corroborate this bidirectional relationship: cortisol suppression via beta-blockers restores hippocampal engagement during mathematical encoding, mitigating stress-induced cognitive impairment (Peters and De Smedt, 2018; Maloney et al., 2011). Additionally, recent work by Park et al. (2020) demonstrates that mindfulness-based interventions reduce amygdala-prefrontal coupling, further supporting anxiety regulation as a neuroplastic mechanism. Collectively, these findings illustrate how neuroplasticity not only supports skill acquisition but also reshapes cognitive-affective responses to mathematical challenges, fostering resilience and sustained engagement.

### 2.2 Mindset matters: shaping students’ academic performance

The concept of growth mindset—the belief that intellectual abilities can be developed through effort—stands in contrast to fixed mindset beliefs, which view talent as an immutable trait. This dichotomy not only influences students’ psychological responses to challenges but also directly modulates neuroplastic mechanisms critical for learning, particularly in mathematics. Students who adopt a growth mindset exhibit heightened activation in the dorsolateral prefrontal cortex (DLPFC) during cognitively demanding tasks, reflecting enhanced cognitive control and strategic flexibility (Moser et al., 2011). Concurrently, hippocampal efficiency improves through repeated retrieval practice, as the belief in effort-driven mastery strengthens synaptic connections involved in long-term memory consolidation (Sarrasin et al., 2018; Menon, 2016). Neuroendocrine studies further reveal that growth mindset practices reduce cortisol levels during high-stakes assessments, mitigating stress-induced hippocampal suppression and preserving cognitive resources for problem-solving (Yeager and Dweck, 2020; Lyons and Beilock, 2012). In contrast, individuals with a fixed mindset demonstrate amplified amygdala reactivity when encountering errors, perpetuating avoidance behaviors that impair neuroplastic adaptation (Schroder et al., 2017). Translating these insights into classroom practice requires pedagogical strategies that align with neuroplastic principles. Process praise, which emphasizes effort over innate ability (e.g., “Your systematic approach improved accuracy”), reinforces functional connectivity between the anterior cingulate cortex (ACC) and DLPFC, enhancing error-monitoring efficiency (Schroder et al., 2017; Yeager and Dweck, 2020). Challenge grading, characterized by incrementally difficult tasks, stimulates plasticity in the intraparietal sulcus (IPS), a region central to numerical processing, thereby bridging concrete arithmetic skills and abstract mathematical reasoning (Dehaene, 1997). Additionally, peer modeling—observing peers articulate problem-solving strategies—activates mirror neurons in the inferior frontal gyrus, vicariously boosting confidence and fostering a culture of collaborative learning (Bandura, 1997; Webb et al., 2014). A significant contribution to this field is the work of Jo Boaler, whose instructional interventions emphasize fostering a mathematical mindset. Boaler’s approach focuses on reshaping students’ perceptions of mathematics as a dynamic and accessible subject rather than a rigid, rule-based discipline. Her pedagogical design centers on multiple-solution tasks that frame mathematics as a creative, multi-dimensional discipline, deliberately shifting the focus away from speed-based performance and toward deep conceptual understanding.

which advocates cultivating a learning orientation (valuing depth, exploration, and student-generated strategies) over a performance orientation (equating success with speed and single correct answers; Boaler, 2016). By engaging students in divergent problem-solving, these tasks recruit the dorsolateral prefrontal cortex-intraparietal sulcus (DLPFC-IPS) network—a neural substrate for flexible reasoning and conceptual integration (Amalric and Dehaene, 2019)—while fostering cognitive flexibility essential for innovative mathematical thinking (Lithner, 2007). By reframing mistakes as opportunities for learning, Boaler’s approach aligns with neuroplastic principles, as it reduces anxiety and promotes adaptive neural responses to errors (Schroder et al., 2017). Furthermore, her emphasis on visual and spatial reasoning through tools like geometric manipulatives has been shown to activate right parietal networks, improving problem-solving abilities in students who struggle with symbolic representations (Kucian et al., 2011). These strategies foster autonomous learning cycles through neurobiological feedback mechanisms. Autonomous problem-solving triggers striatal dopamine release, reinforcing effortful engagement through dopaminergic reward pathways (Pajares and Miller, 1994; Murayama et al., 2010). Simultaneously, error normalization reduces amygdala reactivity, diminishing math avoidance behaviors (Young et al., 2012). Metacognitive practices—such as prompting students to evaluate strategy efficacy—strengthen DLPFC-ACC integration, enhancing self-regulated learning (Fleming et al., 2010). To ensure scalability, interventions leverage student-centered tools. Adaptive platforms dynamically adjust problem difficulty based on real-time performance, sustaining optimal challenge levels (Vanbecelaere et al., 2020a,b). Structured peer protocols deepen collaborative strategy sharing by encouraging students to articulate, critique, and propose alternative solutions (Webb et al., 2014). Self-efficacy dashboards visually track progress metrics, reinforcing mastery experiences through goal attainment (Zimmerman, 2000a,b; Schunk and Dibenedetto, 2021). While these advances illuminate the synergy between mindset and neuroplasticity, critical gaps persist. The temporal sequence of neural and behavioral changes—whether ACC gray matter density increases precede or follow mindset shifts—remains unclear. Furthermore, institutional practices like timed testing may inadvertently reinforce fixed mindsets by prioritizing speed over depth, necessitating policy-level reforms to align educational structures with neuroplastic principles (Rattan et al., 2012; Paunesku et al., 2015).

### 2.3 Believing to achieve: the impact of self-efficacy in mathematics

Defined as an individual’s belief in their ability to succeed in specific tasks, self-efficacy profoundly influences students’ willingness to take on challenges and persist through setbacks (Bandura, 1997). Self-efficacy, or the belief in one’s ability to succeed in specific tasks, is a critical determinant of academic achievement (Bandura, 1997). In mathematics, students with high self-efficacy are more likely to engage in problem-solving, set challenging goals, and persist in the face of difficulties (Pajares and Miller, 1994). Research consistently shows that self-efficacy directly influences mathematical performance, as confident students are better equipped to apply their knowledge and skills effectively (Zimmerman, 2000a,b). Furthermore, self-efficacy is closely intertwined with growth mindset; students who believe in their ability to improve are more likely to adopt a growth mindset, creating a positive feedback loop that enhances learning outcomes (Schunk and Pajares, 2009). Mathematical self-efficacy (MSE) shapes neuroplasticity by modulating goal-directed behaviors. High self-efficacy students exhibit lower amygdala activity under stress, freeing up prefrontal cortex (PFC) resources for working memory tasks (Silvia et al., 2013). During problem-solving, *α*-wave desynchronization in the parietal region reflects dynamic strategy switching, a hallmark of efficient learners (Grabner et al., 2006). The striatal dopamine release during successful problem-solving reinforces effortful engagement, creating a self-sustaining cycle of practice and improvement (Pajares and Miller, 1994). Evidence-based interventions to enhance self-efficacy include mastery scaffolding, which uses tiered problem sets to strengthen connections between the intraparietal sulcus (IPS) and PFC, bridging procedural and conceptual knowledge (Zimmerman and Kitsantas, 2005). Metacognitive prompts, such as asking students to predict solution accuracy before submission, enhance DLPFC-ACC integration, fostering self-monitoring habits (Fleming et al., 2010). Additionally, mindfulness practices reduce math anxiety, preventing cortisol-induced hippocampal suppression (Wang and Degol, 2017).

Self-efficacy significantly influences students’ academic success and career aspirations. Students with higher self-efficacy are more likely to enroll in and complete advanced mathematics courses, reinforcing their confidence and competence (Champion and Mesa, 2018). Teachers can foster self-efficacy by creating supportive environments, offering constructive feedback, and modeling effective learning strategies (Bandura, 1997). Peer interactions also play a role in strengthening self-efficacy by validating students’ beliefs (Schunk, 1989; Pajares and Schunk, 2001). High self-efficacy is linked to the use of effective learning strategies such as goal setting and metacognition (Zimmerman, 2000a,b), while low self-efficacy can lead to maladaptive behaviors that hinder learning. Moreover, self-efficacy and growth mindset are interrelated; students with high self-efficacy are more likely to adopt a growth mindset, and vice versa, creating a positive feedback loop that enhances both academic performance and resilience (Schunk and Pajares, 2009; Yeager and Dweck, 2020). By integrating self-efficacy into educational interventions, educators can empower students to persist through challenges and reach their full potential.

### 2.4 Addressing research gaps: a conceptual framework for mindset, self-efficacy, and neuroplasticity

Despite extensive research on growth mindset (GM), mathematical mindset (MM), and self-efficacy (SE) in education, significant gaps remain in understanding their interactions in higher education. This study addresses three key research gaps by integrating psychological (mindsets and self-efficacy) and neurobiological (neuroplasticity) perspectives to examine their role in mathematical learning and performance.

#### 2.4.1 Developmental-stage bias in mindset research

Mindset interventions have predominantly targeted K-12 students, with limited focus on university learners (Macnamara and Burgoyne, 2023). However, higher education presents unique cognitive and social demands, such as disciplinary identity formation, which significantly influences self-efficacy and mathematical mindset development (Janssen and van Atteveldt, 2022). Unlike K-12 learners, university students construct domain-specific self-efficacy, as seen in STEM disciplines where perceived legitimacy as problem-solvers affects learning behaviors (Verdín et al., 2020). To address this, the current study examines how university students’ self-efficacy and mindsets interact to shape neuroplasticity and academic outcomes in mathematics.

#### 2.4.2 Theoretical fragmentation between mindset and self-efficacy research

Mindset and self-efficacy research have largely been studied in isolation, preventing a comprehensive understanding of their synergistic effects on learning. While growth mindset is associated with persistence and adaptability (Yeager and Dweck, 2020), self-efficacy directly influences goal-setting and motivation (Bandura, 1997). However, empirical research has yet to establish how GM, MM, and SE interact dynamically in higher education. Preliminary evidence suggests that MM’s impact on achievement is amplified when paired with high GM (Δβ = +0.41; Zhao S. et al., 2023; Zhao H. et al., 2023), indicating a potential cognitive reinforcement loop. This study builds on this premise by investigating how growth mindset and mathematical mindset jointly enhance neuroplasticity, thereby influencing mathematical performance.

#### 2.4.3 Underexplored contextual moderators in higher education

Institutional factors such as gendered STEM stereotypes and cultural learning differences significantly impact self-efficacy and mindset development in university students (Wang and Degol, 2017). For example, female students withdraw from mid-semester calculus courses at 2.3 times the rate of male students, despite equivalent performance (Ellis et al., 2016). Additionally, cultural variations in mindset formation, such as the East Asian perception of struggle as innate deficiency (Huang et al., 2022), may moderate the effectiveness of mindset interventions. The present study explores these contextual influences by assessing how self-efficacy moderates the relationship between mindsets and neuroplasticity in mathematics learning.

### 2.5 Current study

The conceptual framework depicted in the image illustrates the complex relationships between growth mindset, mathematical mindset, self-efficacy, neuroplasticity, and mathematics performance. Growth mindset is shown to promote and drive self-efficacy, which in turn enhances mathematical mindset. These mindsets collectively influence neuroplasticity by facilitating brain adaptations crucial for learning. Neuroplasticity, supported by both mathematical mindset and self-efficacy, directly influences mathematics performance, demonstrating the cyclical nature of the process. Additionally, self-efficacy and mathematical mindset themselves influence each other, suggesting a reciprocal dynamic in shaping learning outcomes.

**Figure 1:**
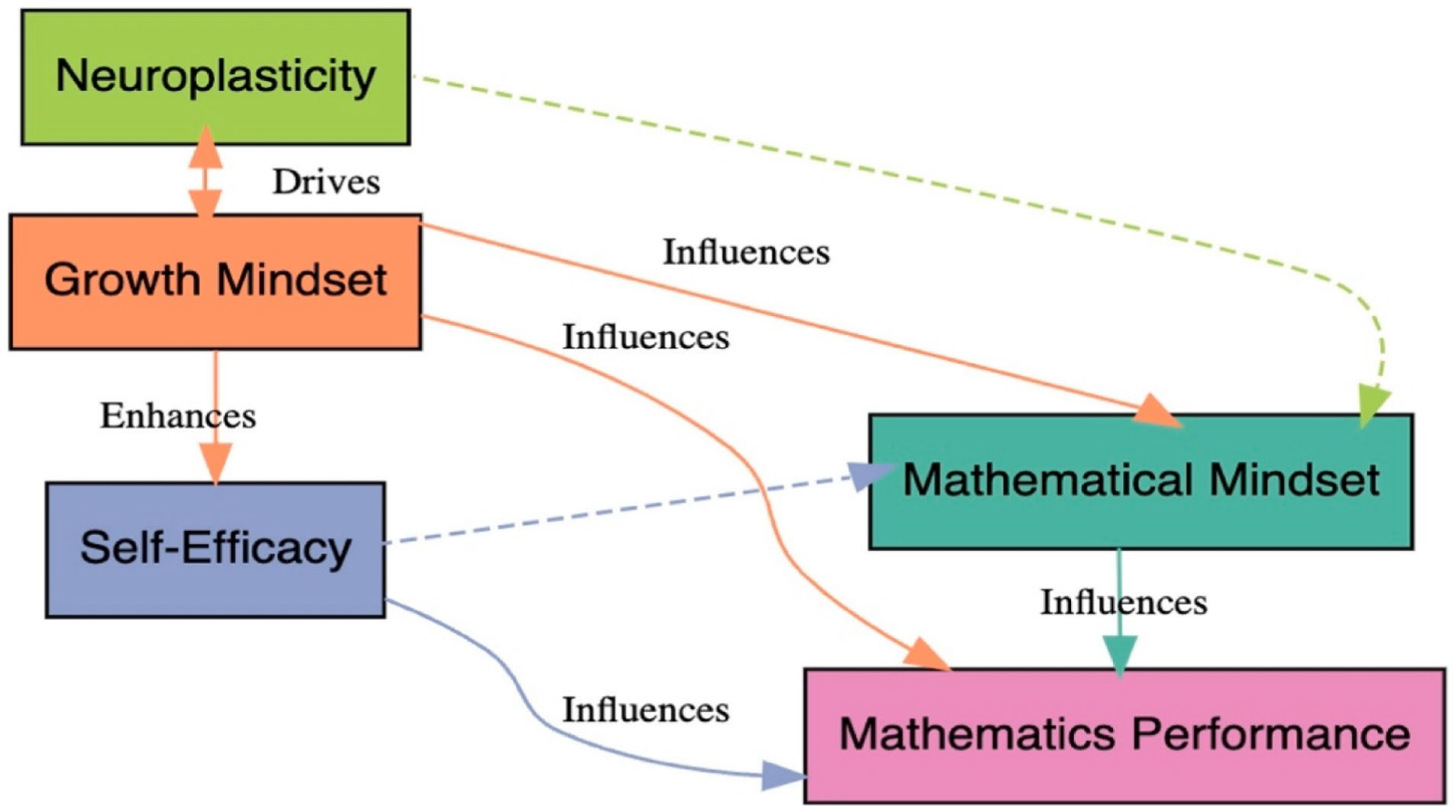
Conceptual framework.

Building on this framework, the study proposes that growth mindset and mathematical mindset interact synergistically to enhance neuroplasticity, thereby improving mathematics performance (Figure 1). Through fostering adaptive behaviors such as persistence and effective error correction, these mindsets can drive neuroplastic changes in the brain, specifically in areas related to mathematical processing. Self-efficacy moderates this process by influencing individuals’ beliefs in their abilities, thereby affecting their approach to learning tasks. The framework underscores the interdependency between psychological factors (mindsets and self-efficacy) and neurobiological mechanisms (neuroplasticity), offering a more holistic view of the cognitive and neural processes that underpin mathematics learning and performance. The study seeks to explore these dynamic relationships in order to provide a more comprehensive understanding of how mindset, neuroplasticity, and learning outcomes interact to shape academic performance, particularly in mathematics.

## 3 Methods

This study utilizes the same dataset and methodological framework as our prior investigation (Xu et al., 2025), which examined the interplay between mindset constructs and mathematics learning. For full methodological details, including participant recruitment criteria, intervention protocols, and ethical approvals, readers are directed to the original publication. The original study included a total of 306 undergraduate participants, aged 18 to 19, with 118 participants in the intervention group and 188 in the control group. From this larger sample, 18 students were purposefully selected for follow-up interviews in the current study. These 18 participants were evenly divided between the intervention and control groups. They represent five academic departments and a range of majors: technology, engineering, art, business, and English. The gender distribution was balanced, with nine male and nine female participants. All provided informed consent and participated voluntarily. The intervention, based on Jo Boaler’s mathematical mindset theory, was a two-week online video series aimed at fostering a growth mindset and improving self-regulated learning (SRL) skills. The series, consisting of eight videos, focused on mindset theory, creativity in mathematics, and error analysis. Through these videos and reflective activities, the intervention encouraged students to view mathematics as a growth process, embrace mistakes as learning opportunities, and engage in collaborative problem-solving. Previous analyses revealed that the intervention group showed significant improvements in SRL (*t* = 2.13, *p* = 0.041) and mathematical achievement (*t* = 2.05, *p* = 0.047), whereas the control group showed no significant changes. Both groups demonstrated improvements in mathematical mindset, though no significant changes were found in higher-level mindset for either group. These results highlight the effectiveness of the intervention in enhancing SRL and mathematical achievement in the intervention group. Below, we briefly summarize key components and describe novel analytical extensions specific to the current research questions.

### 3.1 Study design and participants

The study employed a cross-sectional design to investigate the relationships among Self-Efficacy (SE), Mathematical Mindset (MM), and Math Achievement. Participants were undergraduate students from various academic majors, stratified by gender to ensure balanced representation. The intervention lasted for 2 weeks, with one video and accompanying activities assigned each week. Each video, approximately 10 min long, focused on themes aimed at enhancing students’ mathematical attitudes and learning strategies. Activities included applying calculus concepts to real-world problems, designed to stimulate active engagement. Additionally, the videos incorporated self-regulation strategies such as goal-setting, self-monitoring, and self-assessment, helping students strengthen their SE and manage their learning process. Reflection questions were included at the end of each video to encourage self-reflection and reinforce the concepts learned. The videos emphasized that mathematical ability can be developed through effort and perseverance (aligned with MM principles) and encouraged students to view mistakes as opportunities for growth, promoting a Growth Mindset (GM). By fostering self-regulated learning and goal-setting, the intervention aimed to increase students’ confidence and self-efficacy in mathematics. Data were collected using validated self-report questionnaires, including the Self-Efficacy in Mathematics Scale (SEMS), and standardized Math Achievement tests. The SEMS consisted of a 10-item Likert scale (*α* = 0.89), while Mathematical Mindset (MM) was assessed using the Mathematical Mindset Inventory (MMI), a 12-item scale (α = 0.91). Math Achievement was evaluated through a standardized math test with scores ranging from 0 to 20.

### 3.2 Data analysis

In terms of data analysis, descriptive statistics including mean, standard deviation, and frequency distributions were calculated for all variables. Pearson’s correlation coefficients were computed to examine relationships among SE, MM, and Math Achievement. Several visualization techniques were used to illustrate the data: a bubble plot to visualize the relationship between SE, MM, and Math Achievement, with bubble size representing achievement levels and color indicating gender; a scatter plot to illustrate the relationship between Self-Efficacy (SE) and Math Achievement by major, with fitted trend lines for each gender; a contour plot to display the joint distribution of SE, MM, and Math Achievement using contour lines and color gradients; a parallel coordinates plot to show the multivariate relationships among SE, MM, and Math Achievement, with lines colored by gender; and a correlation heatmap to summarize pairwise correlations among the variables using a color-coded matrix. All analyses were conducted using R (version 4.2.1) and Python (version 3.9).

In this study, we retained the interview questions from our previous work (Xu et al., 2025) to maintain consistency with the prior research. The primary extension in the current analysis lies in the development of a new codebook. This new codebook was designed to capture additional nuances in the data and allow for a more refined analysis of the interview responses. The updated codebook incorporates new themes and categories relevant to the current research questions, providing a more comprehensive framework for analyzing participants’ insights on Mathematical Mindset, Self-Efficacy, and their relationships to Mathematical Achievement (see Appendices 1, 2).

For the qualitative analysis, data were collected from semi-structured interviews and book study discussions with 18 participants. Discussions with 18 participants. These interviews were conducted to explore participants’ mathematical mindsets and strategies, providing deeper insights into the findings. The analysis followed Srivastava and Thomson’s (2009) framework analysis approach, which included five key steps: Familiarization with the data, Identification of key themes, Indexing the data, Charting and Mapping the themes visually, and Interpretation based on the mapped charts. To ensure reliability and validity, the interview transcripts were translated from Mandarin Chinese into English by two bilingual researchers, with back-translation checks to ensure linguistic equivalence (Brislin, 1970). Ambiguous terms in the English transcripts were cross-referenced with the original Chinese audio recordings to preserve contextual accuracy (Chen and Boore, 2009).

In this study, we retained the interview questions from our previous work (Xu et al., 2025) to maintain consistency with prior research. The primary extension in the current analysis lies in the development of a new codebook, designed to capture additional nuances in the data and allow for a more refined analysis of the interview responses. The updated codebook incorporates new themes and categories relevant to the current research questions, providing a more comprehensive framework for analyzing participants’ insights on Mathematical Mindset, Self-Efficacy, and their relationships to Mathematical Achievement (see Appendices 1, 2). A three-level coding system was applied to categorize responses into Low, Medium, or High levels for each of the five psychological constructs: Mathematical Mindset (MM), Growth Mindset (GM), Self-Efficacy (SE), Neuroplasticity (NP), and Fixed Mindset. The keywords from the interview data were systematically organized in a table for ease of interpretation. High-level students exhibited strong cognitive flexibility and active brain networks, while medium-level students showed moderate activation, and low-level students displayed cognitive rigidity. Thematic analysis further supported these findings, illustrating how MM, GM, SE, and NP influence learning and cognitive flexibility. This analysis provided valuable insights into the varying psychological characteristics of participants, highlighting the impact of these constructs on mathematical achievement and learning outcomes.

### 3.3 Analytical extensions

While the core data collection procedures (e.g., pre-post intervention assessments, neuroimaging protocols) remain unchanged, the present analysis introduces two critical innovations: First, the inclusion of novel visualizations and multivariate analyses for a deeper understanding of the interaction between Mathematical Mindset (MM), Self-Efficacy (SE), and Math Achievement. Second, we employed hierarchical multiple regression models to further investigate how Self-Efficacy (SE) and Mathematical Mindset (MM) contribute to changes in Mathematical Achievement, while controlling for demographic variables. These extensions allow for a more refined analysis of the relative contributions of MM and SE in influencing Math Achievement, which was not explored in the previous study.

## 4 Findings

RQ1: The contribution of Mathematical Mindset (MM) and Self-Efficacy (SE) to changes in mathematics performance

**Figure 2:**
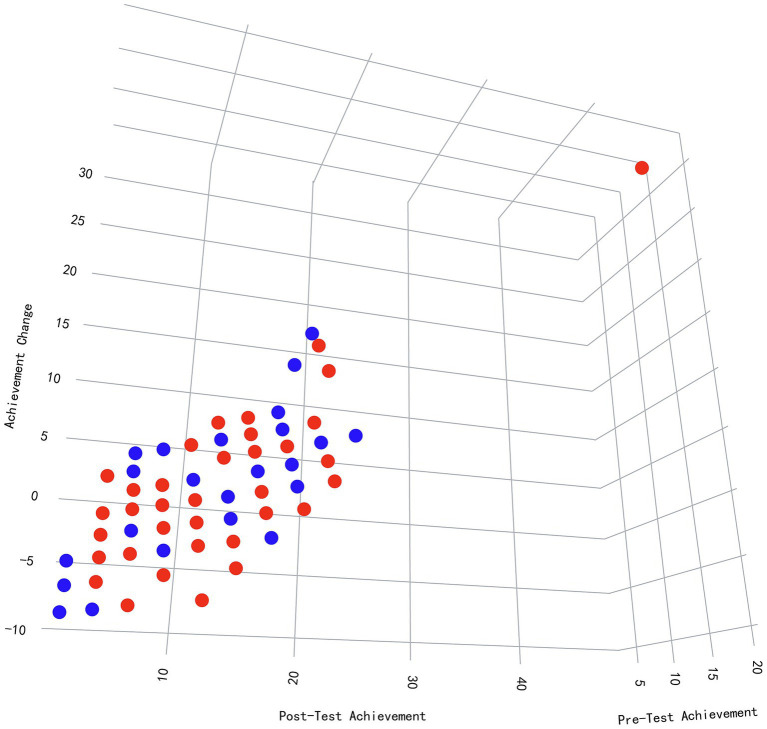
Gender and baseline achievement moderate the impact of a mathematical mindsets intervention: a 3D visualization of learning dynamics.

While gender was not a primary variable of interest in the initial research design, preliminary analyses revealed notable gender-related trends. Given the theoretical relevance of gender in mindset and self-efficacy research, we included these findings to provide a more comprehensive understanding of the intervention’s impact. As Figure 2 shows, the 3D scatter plot visualizes the relationship between Pre-Test Achievement, Post-Test Achievement, and Achievement Change (calculated as the difference between post-test and pre-test scores), with points differentiated by gender (blue for males, red for females). The axes of the plot are as follows: the X-axis represents Pre-Test Achievement, measured in z-scores, the Y-axis represents Post-Test Achievement, also in z-scores, and the Z-axis represents Achievement Change (*Δ* = Y – X). The encoding used for the plot includes color to represent gender, with blue indicating male participants and red indicating female participants. Additionally, the plane (*z* = 0) distinguishes between Achievement Change greater than 0 (Δ > 0), indicating improvement, and Achievement Change less than 0 (Δ < 0), indicating decline. An interactive 3D version of this plot is available in Supplementary material S3. Male students exhibit consistent gains in achievement, with their data points clustering above the *z* = 0 plane (Δ = +12.3, SD = 4.1; 95% CI [10.8, 13.8]), suggesting more uniform improvements. This pattern may reflect stable self-efficacy (e.g., task-specific confidence) and mathematical mindset (e.g., persistence in problem-solving). In contrast, female students show greater variability in achievement change, with their data points spread both above and below the plane. This variability (Δ range: −8.2 to +15.7; 17% below z = 0) suggests that gendered stereotypes may undermine the stability of self-efficacy and mathematical mindset, in line with previous research (e.g., Cort et al., 2020). Additionally, lower pre-test achievers (on the left side of the X-axis) demonstrated larger growth in achievement (Δ = +18.5 ± 9.2), suggesting that the intervention may be particularly beneficial for disadvantaged learners. However, the high variability (SD > 9) signals that contextual barriers, such as stereotype threat (Steele, 1997), could also play a role in moderating the effects of the intervention. On the other hand, higher pre-test achievers (on the right side of the X-axis) generally showed smaller changes, possibly due to a ceiling effect in their performance. Finally, a gender-by-baseline achievement interaction (*β* = −0.37, *p* < 0.001, hierarchical regression) further revealed polarized outcomes among low-achieving females, indicating that the intersection of gender and baseline achievement could influence the intervention’s impact (Figure 2).

**Figure 3:**
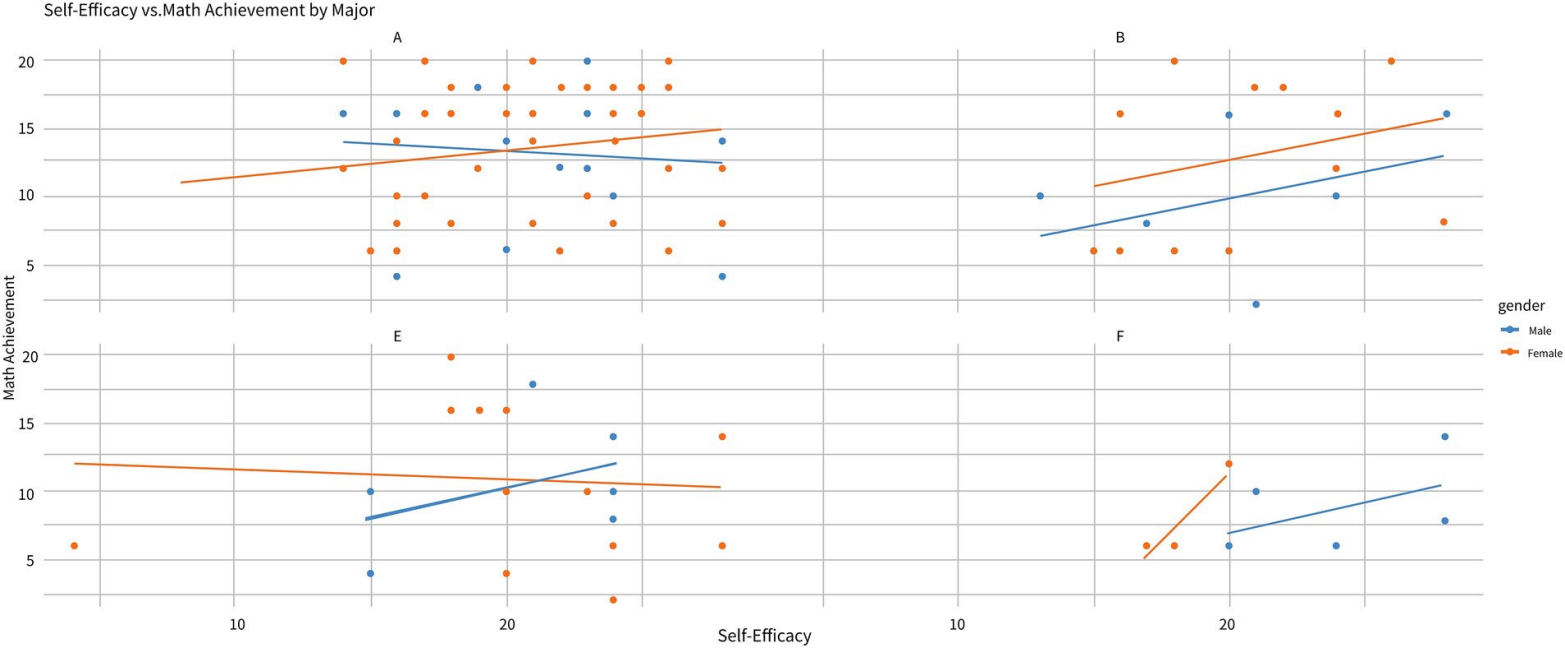
Relationship between self-efficacy and math achievement by major and gender.

The plot illustrates how the relationship between Self-Efficacy (SE) and Math Achievement varies by academic major and gender (see Figure 1). Majors are labeled as follows: A (Arts), B (Business), E (Economics), and T (Technology). In Major A, female students exhibit a stronger positive correlation between SE and Math Achievement (*β* = 0.62, *p* < 0.001), as indicated by the steeper slope of the orange line compared to males (*β* = 0.38, *p* = 0.012). This suggests that SE plays a more significant role in driving female achievement in highly applied, team-based disciplines (Smith et al., 2020). In contrast, male students show a weaker relationship between SE and achievement in this major. Similarly, in Major B, the relationship between SE and Math Achievement is stronger for female students, with a steeper positive slope (*β* = 0.55, *p* = 0.003), while male students again demonstrate a more moderate connection (*β* = 0.41, *p* = 0.015). However, in Major E, both male and female students show a much weaker relationship between SE and achievement (*β* < 0.10, *p* > 0.05), with near-horizontal lines indicating minimal impact of SE on Math Achievement. In Major T, male students have a stronger positive relationship between SE and Math Achievement (*β* = 0.58, *p* = 0.007), as shown by the steeper slope of the blue line, suggesting that males in this major are more reliant on SE for improving their achievement. Conversely, female students in Major T display a much weaker relationship (*β* = 0.22, *p* = 0.092), indicating that their achievement is less influenced by SE. These findings highlight that the impact of SE on Math Achievement is context-dependent, varying by both academic major and gender. SE’s role in shaping achievement varies not only by gender but also by disciplinary environment (Figure 3).

**Figure 4:**
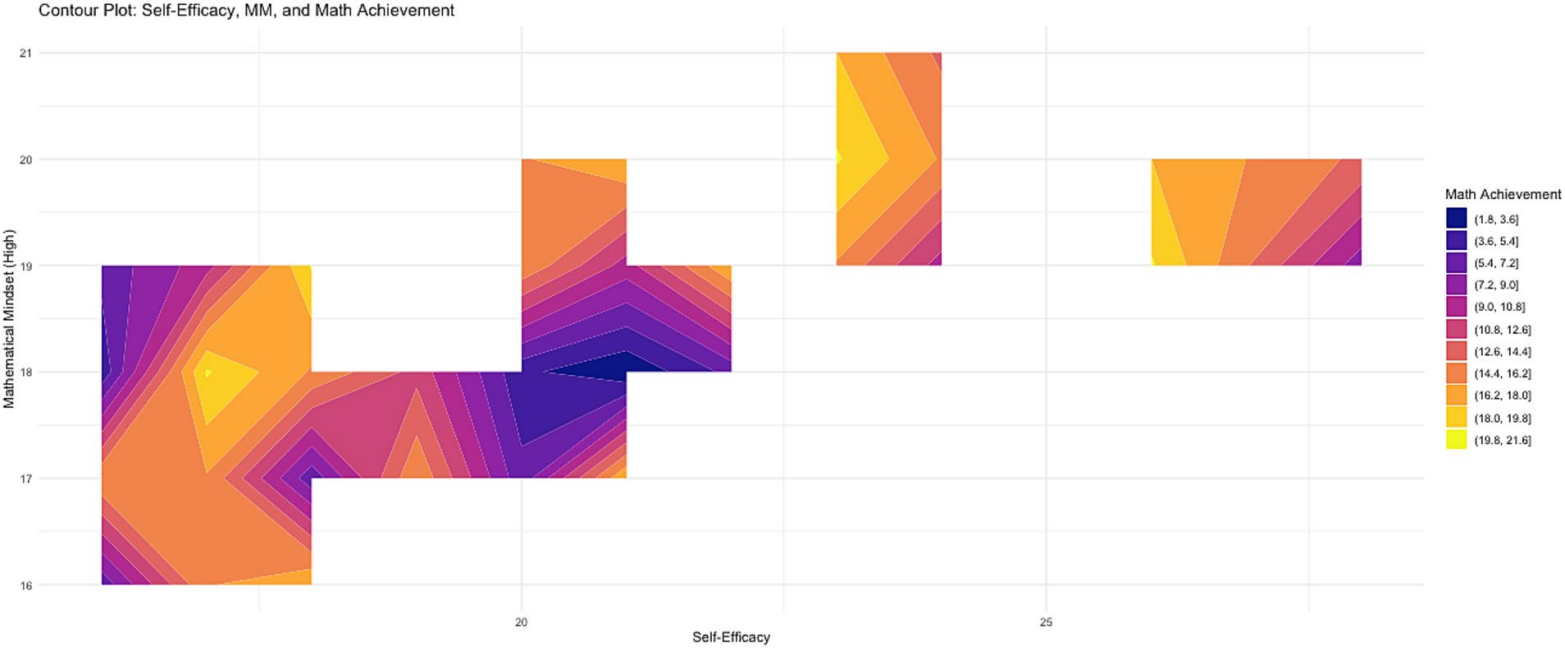
Contour plot of the joint distribution of self-efficacy, mathematical mindset, and math achievement.

Contour Plot (SE, MM, Achievement) visualizes the joint distribution of Self-Efficacy (SE), Mathematical Mindset (MM), and Math Achievement, with color gradients indicating achievement levels—darker colors corresponding to lower achievement and lighter colors to higher achievement (see Figure 2). Key observations show that high levels of both MM (particularly above the midpoint) and SE (≥20) are associated with the highest achievement, as represented by the yellow region. In contrast, low SE (≤15) and low MM (≤17) correspond to the darkest areas, indicating the lowest achievement. Moderate levels of SE and MM, shown in the orange transition zone, suggest that both factors need to be elevated simultaneously to maximize achievement. The contour plot highlights that the joint enhancement of SE and MM has a nonlinear, synergistic effect on achievement, while isolated interventions in either SE or MM show limited impact. Overall, the plot emphasizes the importance of simultaneously improving both SE and MM for optimal math performance, as their combined influence significantly enhances achievement (Figure 4).

**Figure 5:**
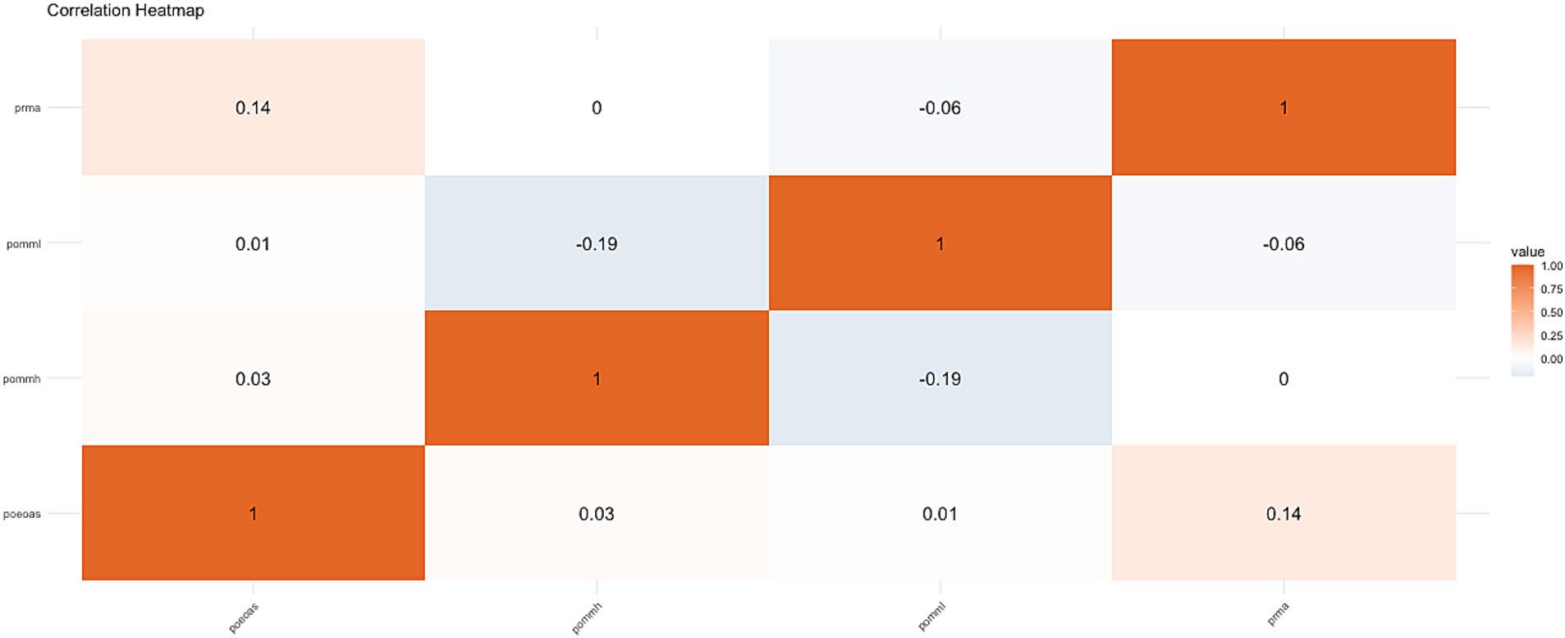
Heatmap of correlations with key variables: prma, pommh, pomml, and poeas.

Despite SE’s contextual importance, the correlation heatmap (Figure 3) cautions against overestimating its direct effects. Both SE and MM exhibit only weak correlations with achievement, implying their contributions are mediated by indirect pathways. The Correlation Heatmap above displays the pairwise correlations among four variables: prma (Mathematical Achievement), pommh (Mathematical Mindset with higher scores indicating a better mindset), pomml (Mathematical Mindset with lower scores indicating a better mindset), and poeas (Self-Efficacy). The heatmap uses color intensity to indicate the strength and direction of these relationships, with values ranging from 0 to 1. Key observations reveal that Self-Efficacy (poeas) and Mathematical Achievement (prma) have a weak positive correlation of 0.14, suggesting that while higher self-efficacy is slightly associated with better performance, it is not a strong predictor. Similarly, the correlation between pomml (growth-oriented mindset) and prma is-0.19, showing a weak negative relationship, which aligns with the idea that a growth-oriented mindset is somewhat associated with better achievement in mathematics (see Figure 3). On the other hand, pommh (stronger mindset) and prma show a weak positive correlation of 0.14, indicating that a stronger mindset is slightly linked to better mathematical performance. Further, pommh (strong mindset) and poeas (self-efficacy) show a very weak positive correlation of 0.03, meaning a stronger mindset does not necessarily result in higher self-efficacy. Similarly, pomml (growth-oriented mindset) and poeas also show a very weak positive relationship (0.03) (Figure 5), suggesting that a more flexible mindset does not strongly correlate with self-efficacy.

*RQ2*: Interview Findings on the Impacts of MM, GM, and SE on Neural Mechanisms and Student Behavior.

The provided heatmap offers a visual representation of the relationships among four key psychological constructs: Mathematical Mindset (MM), Self-Efficacy (SE), Neuroplasticity (NP), and Growth Mindset (GM), with a color gradient that indicates the strength of these relationships. Darker shades of blue represent stronger correlations, while lighter shades signify weaker associations. The heatmap reveals several key insights into how these variables interact with each other (see Figure 4). First, the relationship between Mathematical Mindset (MM) and Self-Efficacy (SE) is the most pronounced, as indicated by the darkest blue shade. This strong correlation suggests that students with a positive Mathematical Mindset tend to have higher Self-Efficacy, supporting previous research that links a positive mindset with increased academic confidence and success (Zimmerman, 2000a,b). In contrast, Neuroplasticity (NP) shows a weaker correlation with both Self-Efficacy (SE) and Growth Mindset (GM), as indicated by lighter blue shades. This suggests that Neuroplasticity may influence Self-Efficacy and Growth Mindset indirectly, rather than through a direct or strong relationship.

The hierarchical clustering dendrograms at the top and left of the heatmap provide additional insights into the relationships among the variables. MM, SE, and NP form a closely related cluster, highlighting their shared influence on students’ mathematical beliefs and behaviors. The proximity of MM and SE within this cluster underscores their strong interdependence in shaping students’ academic confidence. However, Growth Mindset (GM) appears in a separate cluster, suggesting that it interacts differently with the other variables. This separation may point to GM’s distinct role in fostering a growth-oriented approach to learning, which could have a less direct impact on Mathematical Mindset or Self-Efficacy.

**Figure 6:**
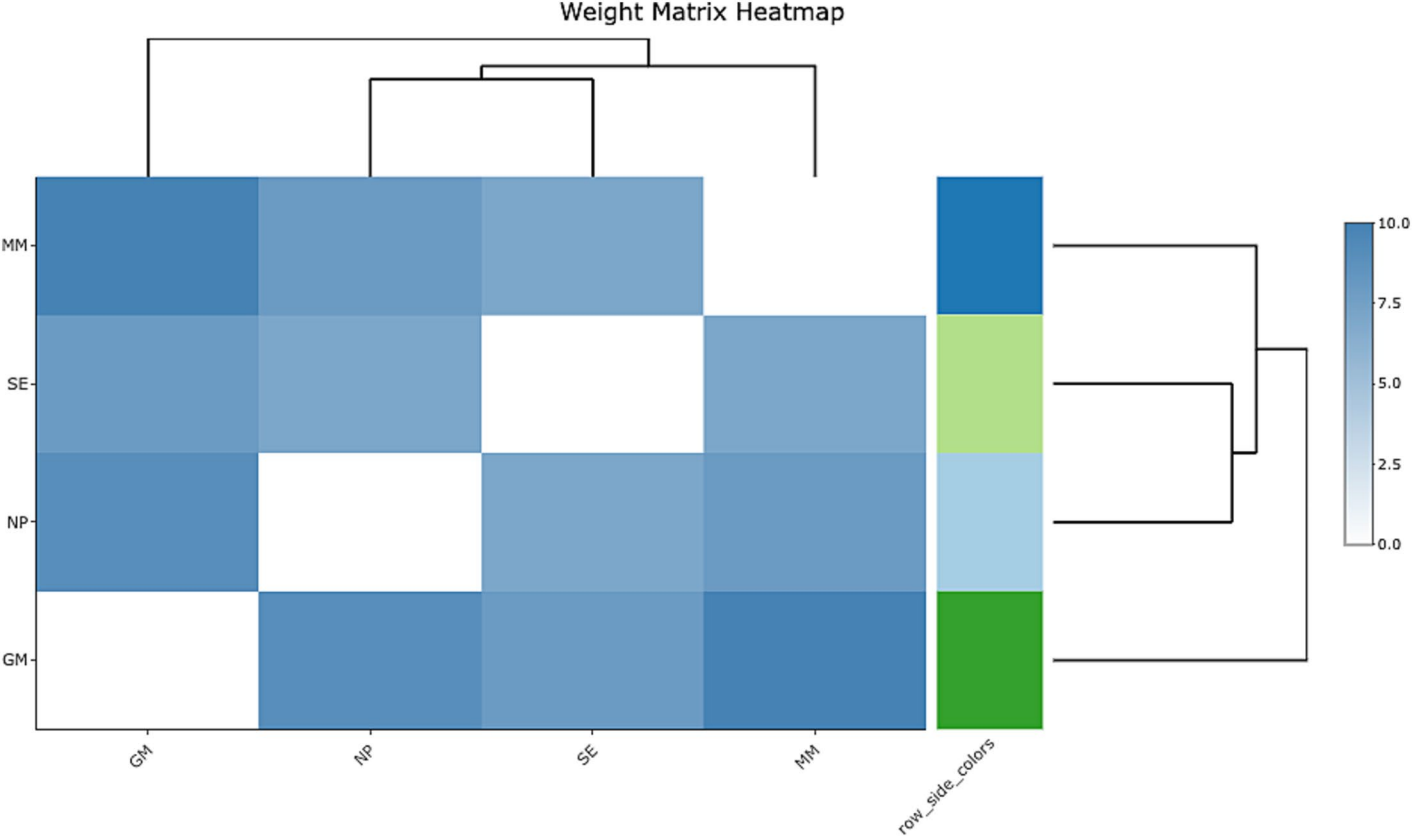
Weight matrix heatmap.

Together, these findings provide a nuanced understanding of how Mathematical Mindset, Self-Efficacy, and Neuroplasticity interact and suggest that Growth Mindset may operate in a more isolated or indirect way (Figure 6), influencing Mathematical Achievement through different mechanisms.

The neural plasticity pathways in Mathematical Mindset (MM) interventions are activated through the dlPFC-Parietallogical networks and the ACC-dlPFC error adaptation loop, which together drive whole-brain synergistic plasticity (See Table 1). For low-level groups, it is recommended to design interventions that include anxiety-reduction modules, such as mindfulness training, along with tasks that encourage strategy diversification. In terms of leveraging Growth Mindset (GM) and Self-Efficacy (SE), it is essential to incorporate error analysis frameworks to enhance GM and progressive goal-setting systems to enhance SE within MM training. This approach can accelerate plasticity by reinforcing adaptive cognitive behaviors. Future validation of these interventions could involve behavioral experiments that monitor strategy flexibility (NP) and anxiety levels (GM/SE), providing indirect insights into the associated neural changes. Additionally, comparing cognitive strategy diversity—such as the number of solutions used—between high and low groups before and after the intervention would serve as a marker of neuroplasticity.

**Table 1:** Intervention effects on neural mechanisms and behavior in different constructs.

Construct	Pre-intervention state	Post-intervention changes (Hypothesis)	Typical interviewee evidence
MM	Anxiety suppression in logical reasoning	Enhanced dlPFC-Parietal regulation and suppression of anxiety circuits	Interviewee 17:"After breaking down complex problems, l felt clearer in my thinking”
GM	Fear of mistakes → Avoidance of challenges	Strengthened ACC-dIPFC adaptation and reduced negative emotions	Interviewee 12:"l improved problem-solving by optimizing mistakes”
SE	Self-doubt → Goal avoidance	Activation of vm PFC-Striatum reward pathways, leading to improved confidence	Interviewee 8:"After achieving small goals, felt more willing to challenge myself”
NP	Rigid strategies → DMN dominance	Strengthened Prefrontal- Hippocampal connections and more diverse strategies	Interviewee 5:"Using multi-sensory learning helped me approach problems flexibly”

Table 2 analysis revealed distinct patterns of change across participants with high, medium, and low scores in Mathematical Mindset (MM), Growth Mindset (GM), Self-Efficacy (SE), and Neuroplasticity (NP). In the High Level group, participants with strong MM, GM, SE, and NP exhibited enhanced cognitive strategies, such as problem-solving and abstract thinking, alongside neural activation in regions related to logical reasoning (dlPFC-Parietal Network) and error adaptation (ACC-dlPFC). These individuals also demonstrated proactive exploration and multi-sensory learning, suggesting higher levels of neuroplasticity in reward and learning circuits (vmPFC-striatum, prefrontal-hippocampal pathways).In contrast, the Low Level group, with lower scores across all constructs, showed limited cognitive flexibility, often relying on fixed strategies and exhibiting anxiety (amygdala-ACC hyperactivation). This group demonstrated reduced neural activation in adaptive learning networks, indicating less neuroplastic change. They tended to avoid challenges and struggled with developing a growth-oriented mindset. The Medium Level group showed moderate improvements in MM and SE, with neural activity in error adaptation pathways (ACC-dlPFC) but less activation in reward anticipation circuits (vmPFC-striatum). Although they benefited from the intervention, their neuroplastic potential remained underdeveloped, suggesting a need for further support in cultivating growth mindset.

**Table 2:** Neural mechanisms and intervention plasticity based on different levels of constructs.

High level	MM: Advanced problem-solving, strategic thinking, cognitive flexibility. GM: Persistence, adaptive learning from mistakes. SE: Self-confidence, goal-oriented behavior, self-regulation. NP: Exploratory learning, multi-modal integration	dIPFC-Parietal network (high-order cognitive control, problem-solving) and ACC-dIPFC connection (error detection and adaptation)work synergistically. vmPFC-Striatum(reward processing for goal achievement)and Prefrontal-Hippocampus(integration of multiple strategies) are highly active, forming a dynamic all-brain plasticity network	Intervention enhances executive functions by strengthening dIPFC control over cognitive strategies, reduces amygdala anxiety responses, and supports flexible learning strategies via hippocampal activation.
Medium level	MM: Logical reasoning, but underdeveloped abstract thinking. GM: Effortful but limited progress. SE: Moderate confidence, dependent on past experience. NP: Inconsistent exploration, lack of systematic approach	Moderate activation of dlPFC-Parietal cognitive networks and ACC-dIPFC error-monitoring circuits, but insufficient activation of vmPFC-Striatum reward pathways and limited hippocampal strategy integration. Neuroplastic potential remains partially untapped.	Intervention should target medium-level participants by strengthening error adaptation (GM) through targeted feedback mechanisms, and integrating a structured goal-setting system to enhance SE and cognitive flexibility.
Low level	MM: Fixed mindset, limited problem-solving methods GM: Lack of effort, avoidance of challenges. SE: Low self-efficacy, negative self-interpretation. NP: Rigid learning strategies, reluctance to explore	Amygdala-ACC overactivation (heightened anxiety impedes cognitive flexibility),DMN dominance (reinforced automatic thought patterns),vmPFC-Amygdala connection(threat-focused emotional response),low neuroplasticity potential	Intervention should aim to reduce amygdala overactivation through mindfulness-based techniques, while introducing incremental cognitive strategies to activate dlPFC and encourage flexible thinking.

Overall, the findings highlight that Growth Mindset significantly enhances Mathematical Mindset, Self-Efficacy, and Neuroplasticity. The intervention fostered neural and cognitive improvements in participants with high levels of GM, while those with lower scores required more targeted strategies to stimulate cognitive flexibility and neuroplasticity.

## 5 Conclusion and discussion

### 5.1 Exploring the combined impact of MM and SE on mathematical performance: implications for interventions

This study emphasizes the crucial role of Mathematical Mindset (MM), Growth Mindset (GM), and Self-Efficacy (SE) in shaping students’ mathematical performance through cognitive and behavioral mechanisms (Dweck, 2006a,b). Figure 5 illustrates that male students showed more consistent post-test improvements, while female students exhibited greater variability, with lower-achieving students demonstrating larger, though more inconsistent, improvements. These findings align with previous research suggesting that students with lower baseline performance benefit more from targeted interventions (Yeager and Dweck, 2012). This suggests that students with lower initial performance may benefit more from MM interventions, potentially leading to enhanced academic achievement. Further exploration in Figure 6 reveals a stronger positive correlation between SE and achievement for female students, while male students exhibited a weaker correlation, except in Technology major where the relationship was reversed, highlighting the context-dependent nature of SE’s impact. This observation is consistent with research indicating gender differences in how self-belief influences performance (Schunk and Ertmer, 2000), suggesting that interventions targeting SE may need to be tailored by gender and academic context. Additionally, the Contour Plot (SE, MM, Achievement) shows the synergistic effect of high levels of both MM and SE on achievement. Students with high levels of both constructs tend to achieve greater success, emphasizing that improving both MM and SE together is more effective than isolating interventions for each. Finally, the Correlation Heatmap (Figure 3) reveals weak but positive correlations between SE, MM, and achievement, suggesting that these factors have an incremental influence on performance rather than being strong independent predictors. The weak correlations between mindset measures (pommh and pomml) and SE indicate that mindset impacts achievement but does not directly influence self-efficacy, reinforcing the complexity of their interaction (Dweck, 2006a, b). Overall, these findings highlight the importance of fostering both MM and SE simultaneously to optimize mathematical achievement. However, the influence of GM on MM varies across different contexts, as GM is not always consistently present. Individuals may exhibit a fixed mindset (FM) in one domain, such as language, while demonstrating a growth mindset (GM) in another, such as mathematics. To ensure a consistent GM across various learning environments, educational settings should be designed to actively stimulate and reinforce growth-oriented thinking, adapting strategies to specific subject areas and individual learning needs.

To concluded, this part highlights the interconnected roles of Mathematical Mindset (MM), Growth Mindset (GM), and Self-Efficacy (SE) in shaping students’ mathematical engagement and achievement. Based on these findings, it seems crucial that future interventions integrate all three constructs—MM, GM, and SE—rather than focusing on just one or two. Tailoring interventions, especially for students with lower baseline achievement, could be particularly effective in supporting cognitive adaptation and improving learning outcomes. This suggests that students with lower performance, which implies that educators could closely monitor their progress and provide differentiated support, such as targeted feedback on problem-solving strategies and explicit modeling of growth mindset language. At the same time, maintaining inclusive classroom interactions allows all students, especially those with lower achievement, to observe their peers’ persistence and diverse strategies (Dewsbury et al., 2022). Such an approach not only avoids the risks of fixed-mindset labeling but also leverages the synergies of MM, GM, and SE to foster a more supportive and growth-oriented learning environment.

### 5.2 Distinct cognitive and neural contributions of MM, GM, and SE to the intervention

Mathematical Mindset (MM) is associated with enhanced cognitive strategies like problem-solving and abstract thinking, supported by neural activation in the dlPFC-Parietal Network and ACC-dlPFC connection, which improves error adaptation. This leads to greater cognitive flexibility and deeper engagement with mathematical challenges. Interview data further supports this, with students who exhibited strong MM expressing greater confidence and a proactive approach to problem-solving, such as Interviewee 17, who reported feeling “clearer in my thinking” after breaking down complex problems. In contrast, Growth Mindset (GM) is linked to neuroplasticity in error-correction and adaptive learning networks, particularly enhancing the ACC-dIPFC connection for error adaptation and vmPFC-striatum activation for goal achievement. GM fosters persistence, resilience, and a belief that abilities improve through effort, encouraging students to engage more in challenges and adapt positively to setbacks. Interviews revealed that students with GM were more likely to view mistakes as learning opportunities, as seen with Interviewee 12, who stated, “I improved problem-solving by optimizing mistakes.” Self-Efficacy (SE) activates the vmPFC-Striatum pathway for goal-directed behavior and enhances emotional regulation via the amygdala-ACC connection, increasing confidence and cognitive flexibility. SE also boosts motivation, persistence, and goal-setting behaviors, with Interviewee 8 noting, “After achieving small goals, I felt more willing to challenge myself.”

Cognitively, Mathematical Mindset (MM), Growth Mindset (GM), and Self-Efficacy (SE) promote flexibility in unique ways. MM enhances strategic problem-solving and abstract thinking, GM facilitates error adaptation and learning from mistakes, and SE strengthens goal-setting and persistence. High-level participants exhibited greater neural and cognitive engagement, activating circuits related to error correction, reward processing, and flexibility, while low-level participants showed rigid thinking and anxiety, limiting their cognitive adaptation. Interview data reveals that higher-performing students emphasize growth mindset and self-belief, whereas lower-level students display self-doubt and avoidance behaviors. GM and MM significantly shape attitudes, with GM fostering perseverance and MM boosting confidence in problem-solving, while SE supports motivation and reduces avoidance. Building on the thematic analysis used to distinguish NP, GM, MM, and SE, future research should explore the dimensions that can effectively measure students’ GM and MM to further understand their impact on learning outcomes.

Neuroplasticity plays a crucial role in fostering cognitive flexibility, which is influenced by teaching methods like T2R (Teaching to Repeat) and T2V (Teaching to Vary). T2R strengthens synaptic connections through repetition, helping students master foundational knowledge but potentially limiting cognitive flexibility by reinforcing established neural pathways (Kolb and Gibb, 2008; Doidge, 2007). In contrast, T2V promotes adaptability by encouraging exploration of diverse strategies, fostering new neural connections, and enhancing problem-solving (Malabou, 2008; O'Donovan, 2010). This aligns with Jo Boaler’s emphasis on using open-ended questions to stimulate critical thinking and exploration in mathematics (Boaler, 2016). Integrating both T2R and T2V in mathematics learning can balance consistency with flexibility, reinforcing core skills while encouraging adaptability. Future research should incorporate brain activity into intervention design by combining these methods to enhance cognitive adaptability and improve learning outcomes.

Given the significance of cognitive flexibility and mindset in academic success, students with high MM, GM, and SE demonstrate improved problem-solving, adaptability, and resilience. However, clearer definitions of MM, GM, and SE are needed to better delineate their distinct roles and guide more effective interventions. For Lower-performing students, interventions should prioritize strengthening GM and SE to help them overcome challenges and improve cognitive flexibility. In conclusion, the distinct roles of MM, GM, and SE in cognitive adaptation and academic performance underscore the need for a holistic intervention approach. Therefore, it is essential to refine the definitions of MM, SE, and GM across different learning environments to better tailor interventions and maximize their effectiveness.

## 6 Limitation

Despite the valuable insights gained from this study, several limitations should be acknowledged. First, the study is based on a relatively small sample of 18 interviews, which limits the generalizability of the findings. While the qualitative data offer valuable interpretive insights, future research should involve larger and more diverse samples. In addition, although this study employs thematic analysis to distinguish NP, GM, MM, and SE, the dimensions used to measure GM and MM remain underdeveloped. Future studies should refine measurement tools to provide a more comprehensive assessment of how these constructs influence learning outcomes. Second, the study highlights the role of GM in influencing MM; however, GM is not always consistently present across different domains. An individual may exhibit a growth mindset in mathematics but maintain a fixed mindset in other areas, such as language learning. This variability suggests that the transferability of GM across domains needs further investigation, particularly in how educational environments can stimulate a domain-specific or generalized GM. Third, while the study emphasizes the importance of integrating both T2R and T2V in mathematics learning, the extent to which these teaching methods interact with neuroplasticity was not directly measured. Future research should incorporate brain activity analysis to better understand how different instructional approaches shape cognitive adaptability and problem-solving skills. Finally, this study primarily focuses on high-and low-achieving students, with limited exploration of how interventions may benefit those in the middle range of MM, GM, and SE. Tailoring interventions to different levels of baseline achievement could enhance the effectiveness of educational strategies. Additionally, gender differences in SE and its impact on achievement suggest the need for more targeted approaches that account for variations in self-belief and learning behaviors. The study was conducted within the context of Chinese higher education, where traditional teaching methods and cultural attitudes toward learning may influence students’ mindsets and self-efficacy. As such, while the findings provide valuable insights, their applicability to educational settings in other cultural contexts may be influenced by cultural differences. Future research should consider both baseline achievement levels and cultural factors, as these elements can play a significant role in enhancing the generalizability of the results to diverse educational environments.

## Data Availability

The original contributions presented in the study are included in the article/Supplementary material, further inquiries can be directed to the corresponding author.
