# Supplementary material for: Differentiating mathematical mindset, growth mindset, and self-efficacy through intervention research: a neuroplasticity approach
Source: Front Psychol. 2025 Jun 5;16:1598817. doi: 10.3389/fpsyg.2025.1598817 (PMC12176877; doi:10.3389/fpsyg.2025.1598817)
Supplement: Supplementary file 1 [file Data_Sheet_1.docx]

**Appendix 1**

| Code/ Theme | Subcode/Subtheme | Coding Indicator | Example Statement |
| --- | --- | --- | --- |
| Fixed Mindset | Belief in innate ability | Believes that people are naturally good or bad at math and that this can't be changed | “You’re either a math person or you’re not. I’m just not a math  person.” |
|  | Fear of failure | Expresses fear of making mistakes or failing in math, views mistakes as negative | “If I make a mistake in math, it means I'm not good at it.” |
|  | Avoidance of Challenge | Tends to avoid or give up on difficult math problems | “When I come across a hard problem in math, I usually skip it or give up.” |
| Growth Mindset | Belief in ability to improve | Expresses the belief that their mathematical abilities  can be developed with effort and practice | “I know I can improve at math if I keep working at it." |
|  | Value of mistakes | Believes that making mistakes is a part of learning  and can lead to improvement | “When I make a mistake in math, I see it as a chance to learn.” |
|  | Persistence | Demonstrates a tendency to persevere in the face of  mathematical challenges | “Even when a math problem is tough, I don't give up. I keep  trying until I solve it.” |
| Mathematical  Mindset | Belief | Expresses believes that everyone can improve their  mathematical skills through learning and practice | “I believe that mathematical skills can be improved through  deliberate practice and continuous learning.” |
|  | Enjoyment of math | Expresses enjoyment or interest in math | “I really enjoy solving math problems. It's like a puzzle to me.” |
|  | Value of math | Recognizes the value of math beyond numbers and its problem-solving benefits | "Math is valuable as it helps develop logical thinking and  problem-solving skills. Anyone can learn and benefit from it." |

**Table 1**

*Codebook*

| Code/ Theme | Subcode/Subtheme | Coding Indicator | Example Statement |
| --- | --- | --- | --- |
| Neuroplasticity | Awareness of Brain  Adaptability | Recognizes that the brain can change and grow  through learning | Practicing math can rewire your brain over time. |
|  | Linking Effort to Brain  Growth | Associates effort/practice with physical brain changes. | Struggling with math helps my brain build new connections. |
|  | Challenges as  Strengthening | Views difficult tasks as opportunities for brain  development. | Tough problems make my brain stronger. I don’t avoid them  anymore. |
| Self-efficacy | Confidence in Ability | Expresses belief in their capacity to succeed in math  tasks. | I’m confident I can solve most math problems if I focus. |
|  | Task Persistence | Demonstrates willingness to engage with math tasks  despite difficulties. | "Even if I don’t get it right away, I’ll keep working until I do." |
|  | Approach to Challenges | Uses strategies (e.g., breaking down problems) to  tackle math challenges. | "I start by simplifying the problem. That helps me feel in control." |

**Appendix 2**

**Table 2**

**Interview analysis**

| Interviewer | MM | GM | Self-Efficacy | | Neuroplasticity | | MM  Keywords | | GM  Keywords | | Self-Efficacy Keywords | | Neuroplasticity Keywords |
| --- | --- | --- | --- | --- | --- | --- | --- | --- | --- | --- | --- | --- | --- |
| Interviewee 1 | Medium | Medium | High | | Medium | | Logical Thinking,  Mathematical Methods | | Effort, Learning,  Improvement | | Confidence, Achievement,  Experience Accumulation | | Trying New Methods,  Partial Active Exploration |
| Interviewee 2 | Medium | High | High | | High | | Logical Thinking,  Problem Solving | | Effort, Growth,  Refinement | | Confidence, Achievement  Memory, Goal Setting | | Active Exploration,  Multisensory Learning |
| Interviewee 3 | Medium | High | Medium | | Medium | | Logical Thinking,  Abstract Thinking | | Effort, Growth,  Refinement | | Confidence, Experience  Accumulation, Learning Process | | Trying New Methods,  Partial Active Exploration |
| Interviewee 4 | Low | Medium | Medium | | Low | | Fixed Mindset, Lack  of Methods | | Effort, Learning,  Improvement | | Confidence, Experience  Accumulation, Goal Setting | | Reliance on Fixed Patterns,  Lack of Exploration |
| Interviewee 5 | High | High | High | | High | | Logical Thinking,  Problem Solving | | Effort, Growth,  Refinement | | Confidence, Achievement,  Goal Setting | | Active Exploration,  Multisensory Learning |
| Interviewee 6 | Medium | Medium | Medium | | Medium | | Logical Thinking,  Mathematical Methods | | Effort, Learning,  Improvement | | Confidence, Experience  Accumulation, Goal Setting | | Trying New Methods,  Partial Active Exploration |
| Interviewee 7 | Low | Low | Low | | Low | | Fixed Mindset, Lack  of Methods | | Lack of Effort,  Fixed Thinking | | Lack of Confidence,  Negative Reactions | | Reliance on Fixed Patterns,  Lack of Exploration |
| Interviewee 8 | Medium | High | High | | High | | Logical Thinking,  Problem Solving | | Effort, Growth,  Refinement | | Confidence, Achievement,  Goal Setting | | Active Exploration,  Multisensory Learning |
| Interviewee 9 | High | High | High | | High | | Logical Thinking,  Abstract Thinking | | Effort, Growth,  Refinement | | Confidence, Achievement,  Goal Setting | | Active Exploration,  Multisensory Learning |
| Interviewer | MM | GM | | Self-Efficacy | | Neuroplasticity | | MM  Keywords | | GM  Keywords | | Self-Efficacy Keywords | Neuroplasticity Keywords |
| Interviewee 10 | Medium | Medium | | Medium | | Medium | | Logical Thinking,  Mathematical Methods | | Effort, Learning,  Improvement | | Confidence, Experience  Accumulation, Goal Setting | Trying New Methods,  Partial Active Exploration |
| Interviewee 11 | Low | Medium | | Low | | Low | | Fixed Mindset, Lack  of Methods | | Effort, Learning,  Improvement | | Lack of Confidence,  Negative Reactions | Reliance on Fixed Patterns,  Lack of Exploration |
| Interviewee 12 | Medium | High | | High | | High | | Logical Thinking,  Problem Solving | | Effort, Growth,  Refinement | | Confidence, Achievement,  Goal Setting | Active Exploration,  Multisensory Learning |
| Interviewee 13 | High | High | | High | | High | | Logical Thinking,  Abstract Thinking | | Effort, Growth,  Refinement | | Confidence, Achievement,  Goal Setting | Active Exploration,  Multisensory Learning |
| Interviewee 14 | Medium | Medium | | Medium | | Medium | | Logical Thinking,  Mathematical Methods | | Effort, Learning,  Improvement | | Confidence, Experience  Accumulation, Goal Setting | Trying New Methods,  Partial Active Exploration |
| Interviewee 15 | Low | Low | | Low | | Low | | Fixed Mindset, Lack  of Methods | | Lack of Effort,  Fixed Thinking | | Lack of Confidence,  Negative Reactions | Reliance on Fixed Patterns,  Lack of Exploration |
| Interviewee 16 | Medium | High | | High | | High | | Logical Thinking，  Problem Solving | | Effort, Growth,  Refinement | | Confidence, Achievement,  Goal Setting | Active Exploration, Multisensory Learning |
| Interviewee 17 | High | High | | High | | High | | Logical Thinking,  Abstract Thinking | | Effort, Growth,  Refinement | | Confidence, Achievement,  Goal Setting | Active Exploration,  Multisensory Learning |
| Interviewee 18 | Medium | Medium | | Medium | | Medium | | Logical Thinking,  Mathematical Methods | | Effort, Learning,  Improvement | | Confidence, Experience  Accumulation, Goal Setting | Trying New Methods,  Partial Active Exploration |

**Appendix 3：Key Constructs and Definitions**

- Mathematical Mindset: This refers to an individual's beliefs and attitudes towards mathematics, including their confidence in learning and performing mathematical tasks. It encompasses the person's perspective on the nature of mathematics and their ability to develop mathematical skills and understanding (Boaler, 2016).
- Growth Mindset: A growth mindset represents the belief that one's abilities and intellectual capacity can be developed and expanded through continuous effort and active learning. This contrasts with a fixed mindset, which maintains that abilities and intelligence are static and unchangeable traits (Dweck, 2006). A growth mindset thus advocates for the possibility of ongoing intellectual growth and personal development.
- Implicit Theory: This term refers to the underlying, unspoken beliefs about the nature of abilities and intelligence. These beliefs shape an individual's motivation and behavior patterns, influencing whether they adopt a growth-oriented mindset (which sees potential for intellectual development) or a fixed mindset (which views these traits as constants) (Dweck, 2006).
- Self-Regulated Learning Strategies: These are deliberate and proactive steps taken by individuals to manage their own learning process. Such strategies include setting clear goals, monitoring progress towards these objectives, and actively seeking feedback for continuous improvement (Zimmerman, 2002).
- Self-efficacy: This concept pertains to an individual's belief in their ability to succeed in specific situations or tasks. Self-efficacy can greatly influence a person's motivation, behavior, and resilience when facing challenges (Bandura, 1977). It plays a significant role in the strategies a person employs to achieve goals and their determination to overcome obstacles.
- Mathematics Achievement: This term denotes an individual's level of performance and proficiency in mathematical skills and knowledge. It is commonly assessed through standardized tests and can be influenced by factors like mathematical mindset, self-efficacy, and self-regulated learning strategies (Boaler, 2016).
- **Contour Plot**
  A contour plot is a two-dimensional graphical representation that displays lines connecting points of equal value, often used to illustrate the topography of a surface or the distribution of a variable across a plane.​
- **Heatmap**
  A heatmap is a data visualization tool that uses color gradients to represent the magnitude of values in a two-dimensional matrix, allowing for quick identification of patterns, correlations, and anomalies within the data.​
- **3D Visualization**
  3D visualization involves creating three-dimensional graphical representations of data, enabling the exploration and analysis of complex datasets by adding depth and perspective to traditional two-dimensional plots.​
